# Assessment of water quality and microbial contamination in Santa Marta’s major rivers using conventional methods and next-generation sequencing

**DOI:** 10.1007/s11356-026-37784-y

**Published:** 2026-05-16

**Authors:** Miguel Mateo Rodríguez, Vanessa Urrea, Nicolas Luna, Isaac Romero, Daniela Palma Polo, Luz H. Patiño, Juan David Ramírez, Marina Muñoz, Lyda R. Castro

**Affiliations:** 1 Centro de Genética y Biología Molecular, Grupo de Investigación Evolución, Sistemática y Ecología Molecular, Universidad del Magdalena, Santa Marta, Colombia; 2 Facultad de Ciencias Básicas y Biomédicas, Maestría en Genética, ADAPTIA, Universidad Simón Bolívar, Barranquilla, Colombia; 3 Centro de Investigaciones en Microbiología y Biotecnología, School of Sciences and Engineering, Universidad del Rosario, Bogotá, Colombia; 4 Grupo Investigación Suelo Ambiente y Sociedad, Universidad del Magdalena, Santa Marta, Colombia; 5 Center for Global Health and Interdisciplinary Research, USF Genomics Program, Department of Global, Environmental and Genomic Health Sciences, College of Public Health, University of South Florida, Tampa, FL USA; 6 Instituto de Biotecnología-UN (IBUN), Universidad Nacional de Colombia, Sede Bogotá, Colombia

**Keywords:** Microbial communities, Water quality, Next-generation sequencing (NGS), Fecal contamination, Sewage

## Abstract

Water quality assessment is critical for determining the suitability of water resources for human consumption. In Santa Marta, northern Colombia, limited access to potable water and sewage systems, along with poor treatment infrastructure, poses a significant risk to both urban and rural communities. In some areas, untreated water is sourced directly from rivers or cisterns. Despite being a public health concern, molecular surveillance of waterborne pathogens remains limited. In this study, we integrated next-generation sequencing (NGS) with conventional physicochemical and microbiological analyses to assess water quality in the Gaira and Manzanares rivers. A total of 24 water samples were collected from eight sites along these rivers between August 2023 and February 2024. Microbial communities were characterized using Oxford Nanopore sequencing targeting the 16S-rRNA and 18S-rRNA genes. Physicochemical variables evidenced high levels of contamination, as elevated concentrations of nitrates, phosphates, ammonium, BOD, and COD were detected across the sampling sites. Other parameters, such as pH and temperature, remained stable, while conductivity and dissolved oxygen varied across sites. Microbiological analyses indicated elevated coliform levels, and *Cryptosporidium* spp. DNA, but not *Giardia intestinalis* DNA, was detected by PCR. Sequencing analysis revealed 1080 prokaryotic and 149 eukaryotic taxa, including environmental bacteria, *Escherichia-Shigella* group, and important taxa like *Enterocytozoon*. Diversity analyses showed that prokaryotic community structure was significantly influenced by basin location (upstream vs. downstream), whereas eukaryotic communities were primarily structured by river identity. Overall, integrating NGS with conventional methods provided a comprehensive characterization of microbial diversity and contamination risks in these tropical river systems, highlighting the urgent need to strengthen water management and public health strategies in Santa Marta and similar regions.

**Supplementary information:**

The online version contains supplementary material available at 10.1007/s11356-026-37784-y.

## Introduction

Water is an essential resource for drinking, recreation, and agriculture, and its quality is closely linked to public health, particularly due to the risk of waterborne diseases (Motlagh and Yang 2019). Assessing water quality is therefore critical for ensuring the safety of water sources (WHO 2017). Major contributors to water contamination include agrochemicals, industrial waste, and human sewage, which frequently lead to microbial pollution, especially in areas lacking adequate water treatment infrastructure (Bartram and Cairncross 2010). This issue is particularly acute in developing countries, where rapid urbanization and the expansion of informal settlements near water bodies intensify human impacts on freshwater systems (Shemer et al. 2023). Consequently, these regions face increasing challenges in maintaining water quality and safeguarding public health.

Conventional water quality assessment relies on the evaluation of organoleptic properties (including color and odor) and physicochemical parameters, such as pH, temperature, turbidity, dissolved oxygen concentration, and suspended solids (EPA 2024). These indicators are essential for characterizing the physical and chemical status of water sources and identifying potential health risks (Rahman et al. 2021). In addition, traditional microbiological analyses, such as total and fecal coliform counts, are widely used as indicators of fecal contamination (EPA 2024). Routine monitoring of these factors enables timely interventions to reduce public health risks (Ballesteros et al. 2023; Torres et al. 2009). However, these methods have important limitations, including limited specificity, long processing times, and restricted capacity to detect a wide range of microbial contaminants. These constraints highlight the need for more sensitive and comprehensive approaches, such as molecular techniques, to improve water quality monitoring and support more effective public health responses (Motlagh and Yang 2019).

Advances in molecular detection of pathogens have significantly improved the ability to identify waterborne microorganisms with greater specificity, speed, and sensitivity (Khodaparast et al. 2024). Next-generation sequencing (NGS), particularly amplicon-based approaches, targeting the 16S-rRNA gene for prokaryotes and the 18S-rRNA gene for eukaryotes, enables high-resolution characterization of microbial communities (Oon et al. 2023). These technologies provide powerful tools to detect microbial contaminants, assess ecosystem health, and generate baseline data to inform water resource management. Importantly, such insights can support evidence-based policymaking and One Health strategies (Tan et al. 2015).

In Colombia, water resources management faces substantial challenges, particularly in ensuring access to potable water and adequate sanitation. Rural and marginalized urban populations often lack sufficient infrastructure and treatment facilities, limiting access to safe drinking water. In many regions, water is sourced directly from untreated rivers, groundwater, or cisterns, increasing vulnerability to waterborne diseases (Gundry et al. 2004). These challenges are exacerbated by increasing industrialization and urbanization, which contribute to the contamination of water resources, and represent a significant public health concern (Borja et al. 2011).

Recent studies using NGS have demonstrated its effectiveness in identifying microbial contamination patterns in Colombian water bodies. For example, analyses of microbial communities in Bogotá’s wetlands and rivers in Pasto have revealed strong associations between anthropogenic pressures and shifts in microbial diversity, including the presence of pathogenic taxa. These findings highlight the impact of untreated discharges and urban runoff on water quality (Ballesteros et al. 2023; Urrea et al. 2025).

In Santa Marta, a major city in the Magdalena region in northern Colombia, water quality represents a critical concern. The city depends heavily on the Gaira and Manzanares rivers as primary sources of drinking water for both urban and rural populations (Londoño et al. 2017). However, these rivers are increasingly affected by anthropogenic pressures, including human settlements, agrochemical runoff, tourism, and inadequate waste management. Previous studies have documented this deterioration. Borja et al. (2011) identified biochemical oxygen demand (BOD), chemical oxygen demand (COD), nitrites, and coliforms as key factors affecting water quality in the lower Manzanares River. More recently, Palma (2025) reported elevated BOD and COD levels exceeding regulatory limits in both the Gaira and Manzanares rivers. In addition, monitoring conducted by CORPAMAG (2021) has shown that microbiological indicators such as total coliforms and *Escherichia coli* frequently exceed permissible limits, reflecting persistent contamination in these basins. These conditions pose a significant public health threat (Harhay et al. 2010; Pásková et al. 2024; Villamizar et al. 2019). The situation is further compounded by limited sanitation infrastructure and rapid urbanization, emphasizing the urgent need for improved monitoring and management strategies. Given its diverse ecosystems, high biodiversity, and environmental pressures (Thiel and Effler 2011; Barragán et al. 2016; Blanco-Cervantes and Blanco-Cervantes 2023), Santa Marta represents an ideal setting for the application of NGS technologies.

Given these challenges, integrating conventional methods with advanced molecular approaches, such as NGS, is essential. This combined strategy has the potential to significantly enhance water quality monitoring, particularly in regions with complex contamination sources, like Santa Marta. In this study, we evaluated water quality in the Gaira and Manzanares rivers by integrating physicochemical and microbiological analyses with NGS, to characterize microbial contamination patterns and their association with environmental and anthropogenic factors. This approach enabled the characterization of both prokaryotic and eukaryotic communities, the identification of potential pathogenic taxa, and the assessment of spatial and temporal variation. The findings provide critical baseline data to support water safety monitoring, risk assessment, and public health interventions in regions facing increasing environmental pressure and limited water treatment capacity.

## Materials and methods

### Study area and eDNA sampling

The study was conducted in the Gaira and Manzanares rivers, two tropical mountain rivers located on the northwestern slopes of the Sierra Nevada de Santa Marta (SNSM), Magdalena Department, northern Colombia. The main channels of these rivers extend for 32.53 km and 33.50 km, respectively, and traverse a variety of ecosystems along their course, ranging from very humid forests to tropical thorn forests, before discharging into the Caribbean Sea and crossing both rural and urban areas of the city of Santa Marta (Borja et al. 2011; Blanco-Cervantes and Blanco-Cervantes 2023). The region is characterized by a tropical climate with a marked bimodal rainfall regime, with rainy seasons typically occurring between April–May and September–November, and dry seasons from December to March and from June to August. The average annual relative humidity is 75% in the mountainous area and 60% in the flat area (Guáqueta-Solórzano and Postigo 2022).

Along their courses, the rivers are influenced by contrasting land uses. Upper and midstream sections are predominantly associated with natural forest cover and agricultural activities, particularly coffee plantations, and small-scale farming (Thiel and Effler 2011). Tourism-related activities occur along the entire length of the rivers, although in contrast, downstream sections flow through areas with higher human population density, more concentrated tourism infrastructure, and urban development, where wastewater inputs, solid waste disposal, and recreational activities are more frequent, making them vulnerable to multiple pollutants resulting from anthropogenic activities.

**Fig. 1:**
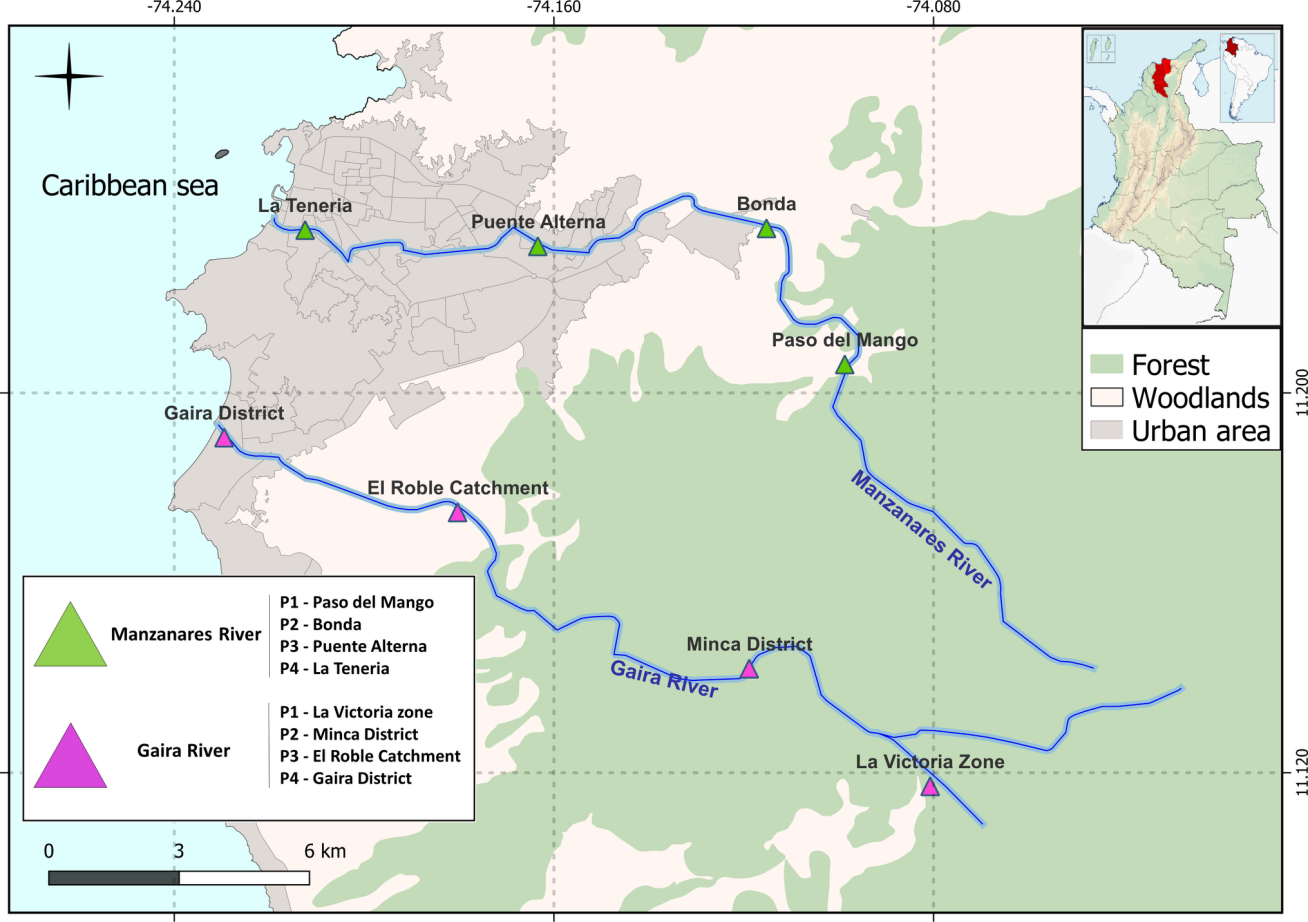
Map with the locations of water sample collection sites in the Gaira and Manzanares Rivers, Santa Marta, Colombia

A total of eight sites were sampled in this study across two rivers: four sites located on the Gaira River (GR) and four on the Manzanares River (MR), distributed across the upper (P1, P2), middle (P3), and lower (P4) basins of each tributary. Sampling was carried out during August (08) and October (10) 2023 and February (02) 2024, for a total of 24 samples (Fig. 1). At each site, a composite water sample was collected by taking 300 mL of surface water every 10 min using a sterile beaker. This process was repeated until a total volume of 900 mL was reached. Samples were stored in sterile bottles and kept in portable coolers with ice until arrival at the Molecular Laboratory of Universidad del Magdalena.

### Traditional physicochemical analysis

Five environmental variables were measured at each sampling site: water temperature, dissolved oxygen, dissolved oxygen percentage, conductivity, and pH. Temperature, dissolved oxygen (mg L^−1^ O_2_), and pH were measured in situ using WTW 3110 multiparameter probes. Additional parameters, including total suspended solids (mg L^−1^), turbidity (UNT), chemical oxygen demand (COD) (mg L^−1^), biochemical oxygen demand (BOD) (mg L^−1^), and nutrients (nitrates, nitrites, phosphates and ammonia (mg L^−1^)) were analyzed at the Water Quality Laboratory of the University of Magdalena, Colombia, using standard analytical methods (Supplementary Table 1). These analyses followed the regulations outlined in Colombian Regulatory Decree 1076 of 2015 (GOV.CO 2023), which applies to recreational waters, agricultural irrigation, and water suitable for human consumption, as well as the World Health Organization (WHO) guidelines (2003) on maximum acceptable risk scores (Supplementary Table 2).

### Traditional microbiological analysis

The microbiological quality of the river water samples was assessed by quantifying indicators such as total and fecal coliforms using the multiple tube fermentation method, SM 9221 B (APHA 2017). Total coliforms were quantified by incubating the samples in Brila broth (Merck, Germany) at 35–37 °C for 24–48 h. After incubation, the tubes that showed gas generation were counted and reported as the most probable number per 100 mL (MPN 100 mL^−1^). For the detection of fecal coliforms, samples were incubated in EC broth (Merck, Germany) at 44 °C for 24–48 h. Similarly, tubes with gas generation were counted and recorded as MPN 100 mL^−1^ (Redondo-Solano and Echandi 2012).

### DNA extraction

DNA was extracted following the method described by Urrea et al. (2025). A total of 600 mL of water per site was vacuum-filtered through 0.45-µm, 47-mm cellulose nitrate membranes and then refiltered using a 0.22-µm, 47-mm membrane to capture smaller microorganisms. A total of two membranes of 0.45 µm and two membranes of 0.22 µm were used to filter 600 mL of water. The membranes were stored in sterile 50-mL Falcon tubes, with the adhered biomass. To recover the biomass, we followed a standardized and previously published protocol (Urrea et al. 2025), which has been successfully applied in similar aquatic metabarcoding studies. All samples were processed using the same procedure, ensuring methodological consistency across sites. Specifically, 15 mL of molecular-grade water was divided among the membranes, and two washes were performed for each membrane. The estimated volume of water was used to scrape the surface of the membrane with the aid of a micropipette for 10 min. After resuspending the biomass, the 15 mL of water was stored in a 15-mL Falcon tube. Subsequently, the sample was centrifuged at 15,000 × g for 10 min, and the supernatant was carefully discarded, leaving only the pellet. DNA extraction was performed from this pellet using the DNeasy PowerLyzer PowerSoil Kit (QIAGEN) according to the manufacturer’s instructions, with an additional chloroform/isoamyl alcohol 24:1 step in the lysis phase to aid phase separation. The extracted DNA was stored at −30 °C.

### Primer selection and PCR amplification

The presence of the genetic material of *Giardia intestinalis* and *Cryptosporidium* spp. was evaluated through the amplification of the 18S-rRNA gene using the primers 5′-CATGCATGCCCGCTCA-3′ and 5′-AGCGGTGTCCGGCTAGC-3′ (87 bp) for *G.* *intestinalis* (referred to as 16S-rRNA in early *Giardia* studies) (Mejia et al. 2013) and CcF18s 5′-GTTTTCATTAATCAAGAACGAAAGTTAGG-3′ and CcR18s 5′-GAGTAAGGAACAACCTCCAATCTCTAG-3′ (107 bp) for *Cryptosporidium* spp. (Burnet et al. 2013) using conventional PCR. The amplifications were performed in a volume of 25 μl with: 12 μl of 2× PCR SuperMix (Bio-Helix, New Taipei City, Taiwan), 1 μl of each primer (10 pmol), 8 μl of ddH2O, and 3 μl of DNA. Negative controls were included in each PCR run. Amplification conditions were as follows: initial denaturation at 95 °C for 5 min, followed by 35 cycles at 95 °C for 40 s, annealing at 58 °C for 30 s, extension at 72 °C for 1 min, and a final extension at 72 °C for 5 min. PCR products were visualized on a 2% agarose gel to confirm the expected amplicon size.

Additionally, the prokaryotic communities were evaluated by NGS using the primers 27 F 5′-AGAGTTTGATCMTGGCTCAG-3′ and 1492R 5′-GGTTACCTGTTACGACTT-3′, which amplify a ~ 1500-bp fragment corresponding to the V1–V9 region of the 16S-rRNA gene (Cuscó et al. 2018). The eukaryotic microbial community was evaluated by NGS using the universal primers 566-F 5′-CAGCAGCCGCGGTAATTCC-3′ and 1289R 5′-ACTAAGAACGGCCATGCACC-3′, which amplify a ~ 750-bp fragment of the V4–V5 region of the 18S-rRNA gene (Hadziavdic et al. 2014).

PCR reactions were performed with the following concentrations: LongAmp Taq 2× Master Mix (New England Biolabs), 200 nM of each primer (10 pmol/μl), and 100 ng of environmental DNA (eDNA). The amplification protocol consisted of initial denaturation at 94 °C for 1 min, followed by 30 cycles at 94 °C for 30 s, 54 °C for 1 min (18S-rRNA)/48 °C for 1:30 min (16S-rRNA), 65 °C for 1 min, and a final extension of 65 °C for 10 min.

### Oxford Nanopore sequencing

PCR products for 16S-rRNA and 18S-rRNA were quantified with the Qubit™ dsDNA HS Assay Kit (Invitrogen, Thermo Fisher Scientific, Waltham, MA, USA) and normalized. Libraries were prepared for sequencing using the MinION platform of Oxford Nanopore Technologies (ONT) following the amplicon protocol of the Native Barcoding Expansion Kit (EXP-NBD106, Oxford Nanopore Technologies, Oxford, UK) and the Ligation Sequencing Kit (SQK-LSK109, Oxford Nanopore Technologies, Oxford, UK). The libraries were then loaded onto MinION SpotON Flow Cells (FLO-MIN106D, R9, Oxford Nanopore Technologies, Oxford, UK) and sequenced for 72 h. A total of 48 samples were processed (four sites per river in three different months), including a library preparation negative control (no-template control), which was processed in parallel with all samples during library construction. Samples were sequenced using the MinION Mk1C device (Oxford Nanopore Technologies, Oxford, UK), with default settings.

### Data analysis

After sequencing, basecalling and demultiplexing were performed using the Guppy Basecaller under the super-accuracy mode and Guppy Barcoder modules of the Guppy tool version 6.3.8 (Wick et al. 2019). During basecalling, low-quality reads with a quality score below 7 were filtered out, and only reads classified as “pass” were retained for downstream analyses (ONT 2021). The quality of the reads was assessed using NanoStat version 1.6.0 with the Nanoplot command (De Coster et al. 2018).

Adapters, primers and chimeric reads were removed using Porechop v0.2.4 (https://github.com/rrwick/Porechop) (Porechop 2018), and Cutadapt v3.5 (Martin 2011). The reads were then trimmed to an optimal length using the seqkit tool version 2.6.0 (Shen et al. 2016) with a minimum length of 600 bp and a maximum of 1500 bp for the 16S-rRNA gene (Jiang et al. 2023) and between 500 and 700 bp for the 18S-rRNA gene (Hadziavdic et al. 2014).

Taxonomic classification was performed using VSEARCH v2.30.0 (Rognes et al. 2016), clustering at 0.97 (Schacksen et al. 2024) and Kraken2 version 2.0.7 (Wood et al. 2019). For Kraken2, a minimum hit group of 3 and a confidence threshold of 0.1 were applied (https://github.com/DerrickWood/kraken2/wiki/Manual) (Lu et al. 2022), with SILVA version 138 for 16S-rRNA (Quast et al. 2013) and the Eukaryotic Pathogen EuPathDB46 database for 18S-rRNA sequences (Aurrecoechea et al. 2017). The data were organized with the Pavian software (Breitwieser and Salzberg 2020). Each sample was processed and sequenced independently and subsequently grouped by sampling point (P1, P2, P3, and P4) within each river for downstream analyses in RStudio version 4.3.2.

### Assessment of microbial communities

To characterize the microbial communities, sequences assigned to Archaea and metazoans were excluded from both the abundance and taxonomic tables using the R package phyloseq v.0.8.12 (McMurdie and Holmes 2013). To visually inspect the total richness of our samples grouped by sampling point (P1, P2, P3, and P4), we generated rarefaction curves using phyloseq and ampvis2 (Andersen et al. 2018). Rarefaction curves were generated to assess sequencing depth across samples. Prior to subsequent analyses, the dataset was rarefied to the minimum sequencing depth across points using the rarefy_even_depth function (rngseed = 1) in phyloseq, in order to minimize biases caused by unequal library sizes, which may affect dissimilarity estimates (Weiss et al. 2017).

A composition analysis was conducted to identify community profiles, where the predominant genera were determined for each river and sampling site based on the relative abundance of the total bacterial community (proportion of reads of each Operational Taxonomic Unit, OTU), using functions from the phyloseq and dplyr packages v1.1.4 (Wickham 2015). The top-most abundant genera were visualized through bar plots using the ggplot2 package v3.5.2 (Wickham 2016). Variations in relative abundances were evaluated through non-parametric statistical tests, specifically the Wilcox tests using functions of phyloseq and dplyr packages. 

Differences in community composition between upstream and downstream sites were tested. Alpha (*α*) diversity was estimated using the Observed richness, Shannon-Wiener, and Simpson indices implemented in the microbiome package of R v.1.30.0 (Lahti et al. 2017). For beta (*β*) diversity, community dissimilarities were explored and visualized with principal coordinate analysis (PCoA) using phyloseq, based on Bray-Curtis distances calculated from relative abundances. In addition, differences in community composition were tested through permutational multivariate analysis of variance (PERMANOVA) with 9999 permutations using the vegan package v.2.7–1 (Oksanen et al. 2015). All visualizations were generated using the ggplot2 package.

To validate genus-level taxonomic assignments of microorganisms potentially associated with pathogenicity, representative sequences were extracted. These sequences were individually queried against the GenBank database using the Basic Local Alignment Search Tool (BLAST) (Altschul and Gish 1996) to identify the closest matches and their corresponding percentage identities. Taxonomic assignments at the genus level were considered when sequence identity was ≥ 95% for both 16S-rRNA and 18S-rRNA genes, with an *E*-value of 0 (Janda and Abbott 2007; Dollive et al. 2012; Srinivasan et al. 2015; Hackmann 2025).

### Canonical correspondence analysis (CCA)

To explore the relationship between microbial community composition and environmental factors, a canonical correspondence analysis (CCA) was performed. The analysis was conducted in R version 4.3.2 using the vegan package (Oksanen et al. 2015) and the phyloseq framework (McMurdie and Holmes 2013). Environmental variables included physicochemical parameters such as nitrites, nitrates, ammonia, phosphates, water temperature, dissolved oxygen, pH, total suspended solids (TSS), biochemical oxygen demand (BOD), chemical oxygen demand (COD), and turbidity (Supplementary Table 1). Variance inflation factors were examined to detect multicollinearity among predictors, and highly redundant variables were excluded to improve model stability. The significance of the global model and individual variables was assessed using permutation-based ANOVA tests (9.999 permutations). In addition, forward selection based on adjusted *R*^2^ (ordiR2step) was applied to obtain a parsimonious model and minimize the risk of overparameterization.

## Results

### Physicochemical analysis

Sites located in areas surrounded by human settlements, particularly in the middle and lower basins (P3, P4) of both rivers, exhibited higher levels of physicochemical alteration. Several variables, including total suspended solids (TSS; 12.34–78.54 mg/L), turbidity (1.45–13.55 NTU), nitrites (21.1–757.5 µg/L), ammonium (2–348 µg/L), phosphates (305.7–2663 µg/L), BOD (4.45–22.76 mg/L), and COD (29.88–74.55 mg/L) presented concentrations above the maximum acceptable limit for water intended for human consumption, according to regulatory standards (Supplementary Fig. 1 and Supplementary Table 2). The pH across all sites ranged from 7.2 to 8.0, while temperatures varied between 18.5 and 29.3 °C. Temperature and conductivity increased with the decreasing altitudinal gradient, whereas oxygen concentrations ranged from 1.4 to 7.74 mg/L (16.4–100.4%) and showed a decreasing trend downstream (Supplementary Table 3).

### Traditional microbiological analysis

**Fig. 2:**
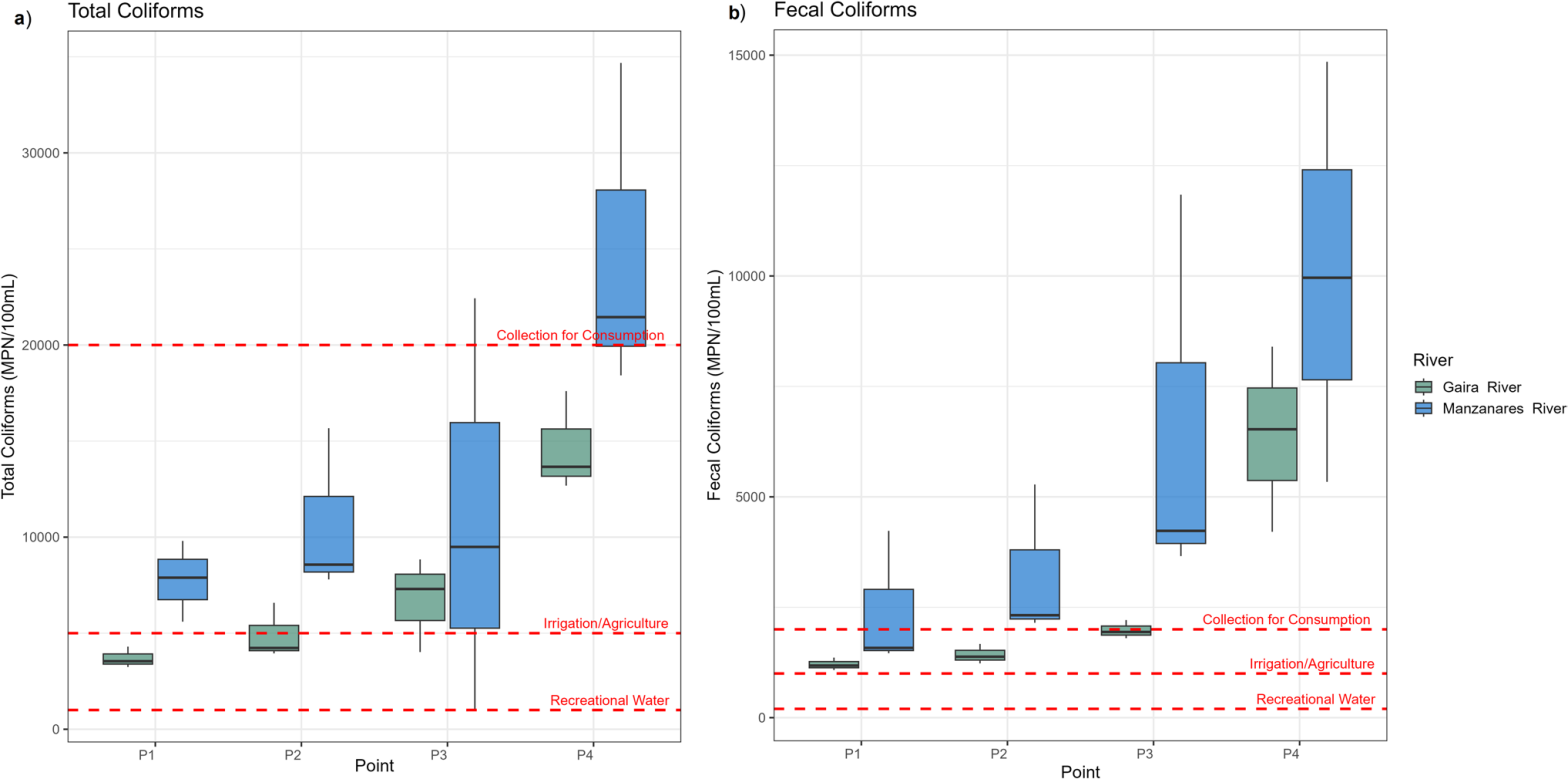
Microbiological quality of water in the Gaira and Manzanares rivers at each sampling site from Aug 2023 to February 2024. **a** Total and **b** fecal coliforms of three independent replicates, measured in MPN 100 mL^−1^. The thick line inside each box indicates the median value, the box represents the interquartile range, and the whiskers indicate the minimum and maximum values. Data are based on three independent replicates per sampling site

Higher concentrations of total and fecal coliforms were recorded in the Manzanares River, reaching 34,680 MPN/100 mL for total coliforms and 14,850 MPN/100 mL for fecal coliforms, whereas the Gaira River exhibited 17,600 MPN/100 mL for total coliforms and 8400 MPN/100 mL for fecal coliforms (Supplementary Table 3). In both rivers, coliform counts increased gradually from upstream (P1) to downstream sites (P4). Additionally, the highest coliform levels were observed in the samples collected during October in both rivers, particularly in the Manzanares River (Fig. 2).

### Detection of genetic material of *Giardia intestinalis *and *Cryptosporidium* spp.

The genetic material of *G.* *intestinalis* was not detected in any of the 24 water samples analyzed. In contrast, *Cryptosporidium* spp. were detected in 9 out of 24 samples (37.5%). Positive detections were recorded during August (*n* = 5) and February (*n* = 4), whereas no positive samples were observed in October. Spatially, *Cryptosporidium* spp. were not detected at upstream sites (P1) in either river. Most detections occurred at midstream (P3) and downstream (P4) sites in both rivers, with an additional positive detection at P2 in the Gaira River during August. No species-level identification was performed; therefore, the results confirm the presence of *Cryptosporidium* spp. at the genus level.

### Analysis of high-throughput sequence data

Of the 24 water samples processed in this study, only one sample, corresponding to the Manzanares River (P3MR_10), was not included in the data analysis, as it generated very few reads after sequencing and did not pass the preprocessing criteria for either the 16S-rRNA or the 18S-rRNA. Rarefaction curves confirmed that the remaining samples had sufficient sequencing depth to accurately assess the diversity and abundance of taxonomic units for both genes (Supplementary Fig. 2 and Supplementary Table 4).

### Description of the prokaryotic communities based on amplicon-based sequencing

For the prokaryotic communities, taxonomic classification attributed most reads to Bacteria. The observed bacterial OTUs were approximately 1080, with the Gaira River showing 899 OTUs and the Manzanares River 949 OTUs after trimming and quality filtering. These OTUs were distributed across 302 genera in the Gaira River and 347 genera in the Manzanares River.

**Fig. 3:**
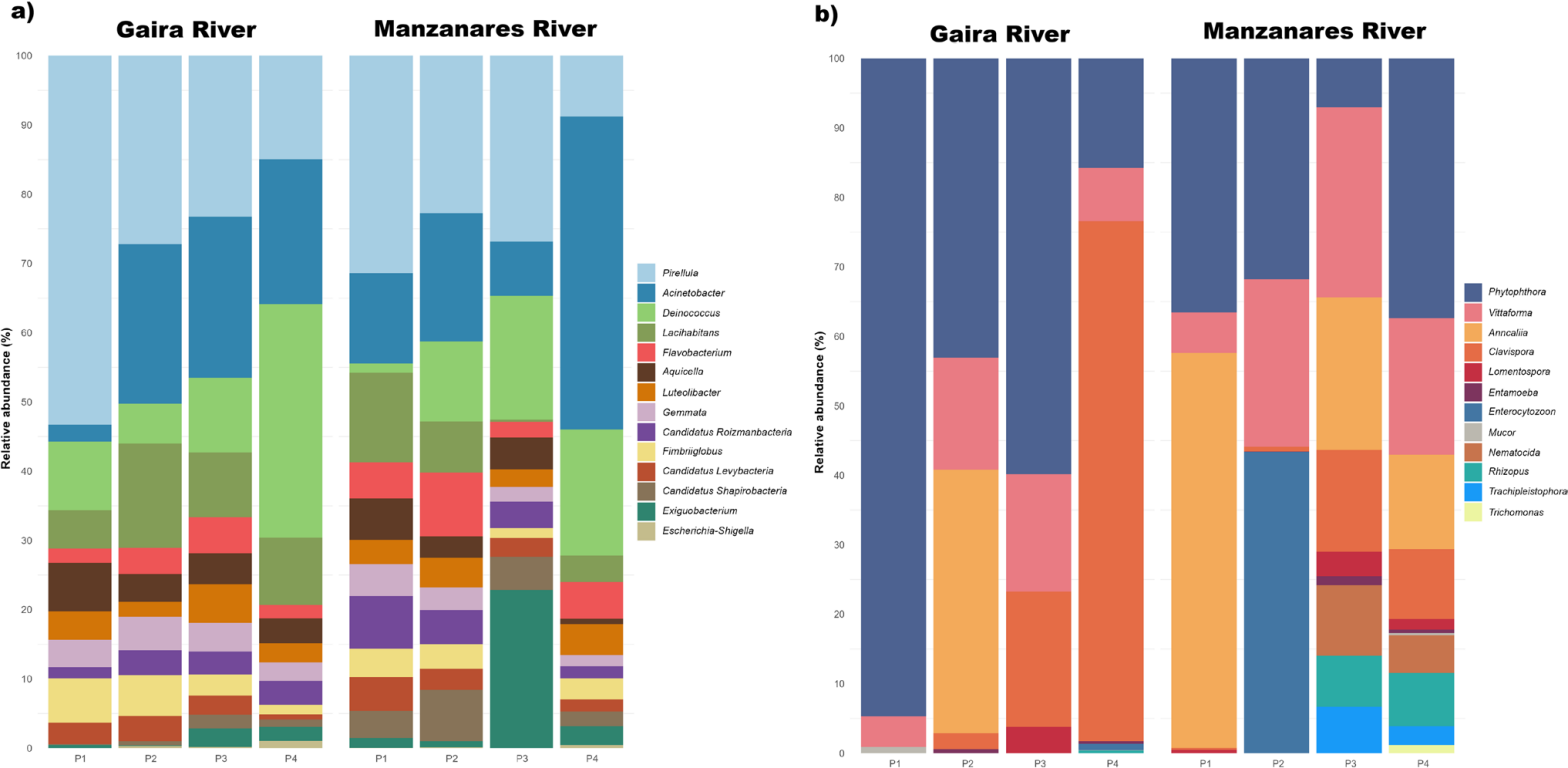
Relative abundance of **a** prokaryotic and **b** eukaryotic genera at four sample points in the Gaira and Manzanares rivers based on amplification of the 16S-rRNA and 18S-rRNA genes. Bars represent mean relative abundances per sampling site derived from triplicate samples. Relative abundances were calculated based on the total bacterial community; only the most abundant genera are displayed for visualization purposes. The genus *Escherichia-Shigella* was retained in the composition plots regardless of its rank, due to its relevance in the study

Overall, both rivers displayed a similar composition of bacterial communities, but a progressive change in their abundances was observed from upstream (P1) to downstream sites (P4) (Fig. 3). The genus *Pirellula* showed a progressive decrease in relative abundance from upstream to downstream locations in both rivers. In contrast, *Acinetobacter*, *Deinococcus*, and *Exiguobacterium* displayed increasing trends toward downstream sites. This pattern was particularly evident for *Exiguobacterium*, which showed a marked increase at P3 in the Manzanares River.

The upstream sites P1 and P2 showed a more uniform distribution of genera, with no single genus strongly dominating the community, whereas the downstream sites P3 and P4 were characterized by an increased relative abundance of specific genera. Additionally, the *Escherichia-Shigella* group was detected at all sampling points, with higher relative abundance at P2 in the Gaira River and P4 in both rivers. BLAST searches of representative sequences assigned to this group returned closest matches to *Escherichia coli*, with sequence identity values ranging from 96 to 97.61% (Supplementary Table 5). These matches represent the closest BLAST hits and do not imply definitive species-level identification.

Additionally, other bacterial genera with potential public health relevance were detected in low abundance in several samples, including *Mycobacterium* (BLAST hits: *Mycobacterium* sp. and *Mycobacterium* *dioxanotrophicus*, 95.71–97.66% identity), *Staphylococcus* (BLAST hits: *Staphylococcus succinus* and *Staphylococcus* *haemolyticus*, 96.35–98.26% identity), *Clostridium* (BLAST hit: Clostridium sp., 95.34% identity), and *Enterococcus* (BLAST hit: *Enterococcus* *bulliens*, 98.86% identity). Again, these matches represent the closest BLAST hits and do not imply definitive species-level identification (Supplementary Table 5).

### Description of the eukaryotic communities based on amplicon-based sequencing

For the eukaryotic communities, a total of 149 taxonomic units were identified at the genus level, with the Gaira River showing 137 OTUs and the Manzanares River 136 OTUs after trimming and quality filtering. The most abundant taxa corresponded to fungal lineages, including *Phytophthora,* *Clavispora*, and *Rhizopus*, together with the microsporidia *Vittaforma*, *Anncaliia*, *Nematocida*, and *Enterocytozoon* (Fig. 3b). Both rivers exhibited a broadly similar community composition, with variations in the relative abundances of certain genera.

Additionally, several groups of sequences show close matches with taxa of potential relevance to human or animal health. These included sequences affiliated with *Enterocytozoon* (with BLAST hit to *E.* *bieneusi*, 95–99% identity), *Entamoeba dispar* (matching *E.* *dispar*, 96.58–98.63% identity), *Rhizopus* *delemar* (matching *R.* *arrhizus*, 95–98.16% identity), *Trachipleistophora* *hominis* (matching *T. hominis*, 94.44–98.80% identity) and *Clavispora lusitaniae* (*C.* *lusitaniae,* 96–98.64% identity). Given the moderate sequence identities and variable query coverage (64–100%) observed in some cases, these assignments should be interpreted cautiously. The species names reported correspond to the closest BLAST matches and do not support species-level identifications. Further BLAST verification revealed that the sequences initially annotated as *Vittaforma* did not consistently match this genus. Instead, they showed identity with various yeast and fungal taxa, suggesting that the original annotation likely resulted from misclassification in the reference database (Supplementary Table 6).

### Alpha and beta diversity analysis

Alpha diversity analyses revealed contrasting patterns between prokaryotic (16S-rRNA) and eukaryotic (18S-rRNA) communities. For the eukaryotic community, no significant differences were detected between upstream and downstream sites in either river for any of the diversity indices (observed, Shannon, and Simpson; Wilcoxon rank-sum tests, *p* > 0.05). In contrast, the prokaryotic community showed significant differences in the Manzanares River for the Shannon and Simpson diversity indices (Wilcoxon rank-sum tests, *p* < 0.05), while the Observed index was not significant. No significant differences were detected for the prokaryotic community in the Gaira River (*p* > 0.05 for all indices; Supplementary Table 7 and Supplementary Fig. 3).

For the prokaryotic community (16S-rRNA), PERMANOVA showed no significant differences between rivers (*p* = 0.4251). However, a significant interaction between basin and longitudinal position (upstream vs downstream; *p* = 0.0117) was detected. In contrast, for the eukaryotic community (18S-rRNA), no significant effect of longitudinal position between basins was observed (*p* = 0.144), suggesting that community composition remained relatively consistent between upstream (P1, P2) and downstream (P3, P4) sites within each river. However, river identity had a significant effect on community structure (*p* = 0.0488), indicating compositional differences between rivers (Supplementary Fig. 4 and Supplementary Table 8).

### Canonical correspondence analysis (CCA)

Given the relatively low sample-to-variable ratio, an exploratory canonical correspondence analysis (CCA) was performed to evaluate the influence of physicochemical variables on microbial community composition. CCA revealed limited explanatory power of the measured physicochemical variables on community structure for both gene markers. The global constrained models were not statistically significant (permutation test, *p* > 0.05), and forward selection based on adjusted *R*^2^ did not retain any predictors in the final models. Although dissolved oxygen exhibited a significant marginal effect, its contribution was insufficient to support a parsimonious constrained model.

## Discussion

Water is essential for life, and its quality directly affects human health. Safeguarding water resources is particularly critical in regions with limited treatment infrastructure. This study combines conventional water quality assessments with next-generation sequencing (NGS) to provide an integrated view of microbial contamination and its environmental and anthropogenic drivers in the Gaira and Manzanares rivers, two rivers from northern Colombia.

### Physicochemical analysis

The physicochemical parameters measured in this study indicate that the Gaira and Manzanares rivers exhibit characteristics typical of tropical mountain rivers, including high dissolved oxygen and near-neutral pH, consistent with previous reports (Serna et al. 2015; Tamaris-Turizo et al. 2013). However, evidence of contamination was observed. Elevated concentrations of nitrates, phosphates, ammonium, BOD, and COD were detected across the sampling sites (P1–P4) (Supplementary Fig. 1). These patterns suggest the influence of anthropogenic activities, including human settlements, agricultural runoff, inadequate waste management practices, and tourism-related pressures, particularly in the lower sections of both rivers (Xu et al. 2022; Zúñiga et al. 1994).

### Coliform contamination in river systems

Coliforms and related bacteria (Enterobacteriaceae, Clostridiaceae) are recognized bioindicators of fecal pollution and are associated with gastrointestinal and other infections (Li et al. 2021; Ríos-Tobón et al. 2017). Traditional microbiological analysis revealed the presence of elevated coliform levels (Decree 1594/1984), showing a clear upstream-downstream gradient (Fig. 2; Supplementary Tables 2 and 3). This pattern was supported by high-throughput 16S-rRNA sequencing, which detected genetic material of the *Escherichia-Shigella* group (Fig. 3a) with higher counts associated with urban settlements and areas of high touristic pressure, such as the district of Minca (P2), Rodadero, Gaira district (P4) in the Gaira River. In the Manzanares River, similar patterns were observed, with higher counts associated with recreational areas (P2) and urban areas of Santa Marta city (P4). Peaks recorded in October coincided with the rainy season (IDEAM 2025), during which increased surface runoff can enhance the mobilization and transport of contaminants derived from anthropogenic sources, including wastewater discharges, solid waste, agriculture, and tourism into the river system. These findings align with previous reports of fecal indicators in human-impacted rivers, underscoring the combined role of land use, urban expansion, seasonality, and inadequate waste management in shaping water quality (Ballesteros et al. 2023; Falco et al. 2021; Fernandes-Pineda et al. 2024; Sara-Lilia et al. 2019; Ramos et al. 2008; Ríos-Tobón et al. 2017). These conditions, together with high nitrates, phosphates, COD, and BOD (Supplementary Fig. 1), limit the suitability of water for recreation, agriculture, livestock, and human consumption without treatment (Borja et al. 2011; Decree 1076/2015).

### Detection of the genetic material of *Giardia intestinalis* and *Cryptosporidium* spp.

Molecular analyses targeting the genetic material of waterborne protozoan pathogens provided additional insight into the potential sanitary relevance of the studied rivers. The absence of *G. intestinalis* detection by both PCR and NGS, together with the failure of NGS to recover *Cryptosporidium* spp. despite their detection by PCR, may be attributed to low DNA concentrations that hindered amplification, or to the presence of *Giardia* species not targeted by the primers used (Chan et al. 2022; Yousefvand et al. 2021). Additionally, the theoretical limit of detection (LOD) associated with the 600-mL filtration volume used in this study may have influenced the detection of microorganisms present at very low abundances. In such cases, non-detection does not necessarily indicate true absence but may instead reflect concentrations below the detection threshold of the sampling volume, as previously discussed for waterborne pathogens (Smith and Nichols 2010).

Previous studies have linked the detection of *Cryptosporidium* in freshwater systems to anthropogenic contamination sources, including domestic wastewater discharges, recreational water use, tourism-related activities, and agricultural runoff associated with livestock (Alves et al. 2018; López et al. 2020; Maurya et al. 2013). Such inputs facilitate the introduction of environmentally resistant oocysts into surface waters, promoting their persistence and potential downstream accumulation. The presence of *Cryptosporidium* therefore represents a potential public health concern, particularly in a region reporting high rates of gastrointestinal infections in Colombia (Rodríguez-Morales et al. 2016; Villalba-Vizcaíno et al. 2018). Future studies should prioritize species-level identification using higher-resolution molecular markers such as *β-giardin*, *gdh*, and *tpi* for *Giardia*, and *gp60* and full-length 18S-rRNA for *Cryptosporidium* to better distinguish zoonotic from anthroponotic transmission pathways and refine epidemiological risk assessments in Santa Marta and the Magdalena Department. 

### Description of the prokaryotic communities based on amplicon-based sequencing

Our taxonomic identification results of prokaryotic communities, based on 16S-rRNA gene sequencing, revealed the dominance of several bacterial genera associated with diverse environmental water niches and the dynamics of these ecosystems (Methé et al. 1998). Many of these taxa are known to participate in key ecological processes such as nitrogen fixation, denitrification, biogeochemical cycling, and the decomposition of organic matter (Alves et al. 2014; Garner et al. 2023; Willems et al. 1991). Genera typically associated with nutrient cycling were more prevalent at upstream sites (P1–P2), reflecting environments with lower anthropogenic pressure, higher photosynthetic activity, and greater oxygen concentration. In contrast, genera displaying increasing trends toward downstream sites are commonly described as metabolically versatile taxa. Many of these organisms exhibit tolerance to environmental stress, capable of degrading a wide range of complex compounds while proliferating under elevated nutrient conditions (Doughari et al. 2011; Pandey 2020). Their increased relative abundance downstream is consistent with the physicochemical gradients observed. This pattern suggests that upstream communities are structured by taxa involved in natural biogeochemical processes, whereas downstream communities reflect the influence of anthropogenic nutrient enrichment and organic matter inputs, particularly from wastewater discharges, tourism, and agricultural activity along the river courses.

In addition to taxa linked to ecological functions and the *Escherichia-Shigella* group (mentioned above), other bacterial genera with potential public health relevance were identified, including *Mycobacterium*, *Staphylococcus*, *Clostridium*, and *Enterococcus*. *Mycobacterium* comprises species pathogenic to humans and animals (Dávalos et al. 2021; Mtetwa et al. 2022) and, although primarily transmitted via the airborne route, its presence in environmental matrices such as water, including drinking water, rivers, and wastewater in Colombia (Bedoya et al. 2022; Dávalos et al. 2021), has raised the possibility of alternative transmission pathways, such as fecal–oral routes, though evidence remains limited (Allen et al. 2021). *Staphylococcus* includes clinically important species responsible for a wide range of infections, from superficial to invasive (Cervantes-García et al. 2014), and it has been detected in aquatic systems and treatment processes (Zieliński et al. 2020). It has also been reported locally in water and food sources (Bedoya et al. 2022; Echeverry-Gallego et al. 2024). Finally, *Clostridium* and *Enterococcus* are commonly used indicators of fecal contamination associated with wastewater discharges and gastrointestinal infections (Moreno et al. 2019); their low abundance in this study likely reflects their typically low environmental concentrations (Suzuki et al. 2012).

### Description of the eukaryotic communities based on amplicon-based sequencing

In terms of the eukaryotic community, the genus *Phytophthora* is of phytosanitary importance, acting as a plant pathogen that causes diseases in crops (Cárdenas et al. 2021; Hansen et al. 2012). *Lomentospora* is primarily saprophytic and is commonly found in crops, irrigation waters, soils, and decomposing organic matter, where it plays a crucial role in nutrient recycling within both terrestrial and aquatic ecosystems (Tiscornia et al. 2009). In contrast, other genera, such as *Anncaliia*, are parasites of invertebrates and planktonic crustaceans and are commonly found in river ecosystems (Sokolova et al. 2020).

However, some genera identified in this study are also of medical relevance. For instance, the detection of genetic material of *Enterocytozoon*, detected through NGS and BLAST hits to *E.* *bieneusi* (95–99% identity), is frequently associated with gastrointestinal infections and is known to infect humans as well as a wide range of wild mammals and birds (Kwon et al. 2021). *E.* *bieneusi* is the most diagnosed in humans worldwide and is primarily associated with chronic diarrhea and wasting syndrome. Its presence in water sources is well documented and is mainly linked to fecal contamination from domestic and wild animals, playing an important role in the epidemiology of this parasite (Dowd et al. 1998). Additionally, *E.* *bieneusi* has been detected in fresh fruits and vegetables intended for human consumption, and the identification of its spores in water supplies suggests that water may serve as a potential transmission route for both humans and animals. This dual host spectrum may explain its detection in both upstream areas, characterized by high biodiversity and wildlife reservoirs, and downstream sites, where dense human settlements, tourism activities, synanthropic hosts, and wastewater discharges may contribute to its accumulation.

In parallel, the presence of genetic material of *Entamoeba* in water sources is strongly associated with fecal contamination. Although the species identified through NGS and BLAST were mostly similar to *E.* *dispar*–like sequences*,* considered non-pathogenic or of low pathogenicity, they are molecularly very similar to *E. histolytica*, the clinically most important pathogenic species within the genus (Bahrami et al. 2019). Given that the identity percentages obtained in the analyses were relatively close among these species, the presence of *E. histolytica* cannot be definitively ruled out.

The genus *Vittaforma* comprises opportunistic fungi that infect a wide range of animals, causing conditions ranging from ocular infections to chronic diarrhea (Chen et al. 2018). Although sequences initially identified as *Vittaforma*-like through NGS did not match reference sequences of this genus upon further verification, several molecular studies have reported a high abundance of microsporidian-related sequences in aquatic environments (Chen et al. 2018; Dowd et al. 1998). This apparent misclassification may be associated with limited representation of certain fungal groups in the reference database, low sequence identity values, and/or partial query coverage, particularly in fungi where commonly used markers such as 18S-rRNA often show limited taxonomic resolution due to their highly conserved nature (Anderson et al. 2003; Radosevich et al. 2025).

### Composition and diversity analysis

Composition and diversity analyses indicated low variability in the microbial assemblage along the upstream-downstream gradient, with only localized shifts in prokaryotic diversity. Comparable freshwater systems have reported similar patterns, where longitudinal position does not always drive strong differences in relative abundance or community composition (Urrea et al. 2025). Despite this, the taxonomic profiles observed are consistent with previous studies describing prokaryotic and eukaryotic communities in freshwater systems (Ballesteros et al. 2023; Garner et al. 2023; Suescun-Sepúlveda et al. 2023; Yang et al. 2019). This apparent homogeneity may reflect a certain ecological resilience of microbial groups, or that these rivers are not yet subjected to levels of pollution severe enough to drastically alter community composition. Nonetheless, both rivers are exposed to comparable pressures from agriculture, intense tourism, and urban activities, and subtle shifts in community structure suggest that distinct physicochemical factors continue to modulate microbial assemblages along the fluvial gradient. Importantly, such environmental pressures may also favor the persistence or emergence of microorganisms of medical relevance, highlighting the potential risks associated with human and animal exposure to contaminated waters. These findings underscore the importance of integrating physicochemical parameters with molecular approaches to better understand the ecological dynamics and health implications of freshwater microbial ecosystems.

While the overall microbial assemblages appeared relatively homogeneous along the longitudinal gradient, the limited taxonomic differentiation observed for certain protozoan and fungal groups may partly reflect methodological constraints. Specifically, some metabarcoding markers lack sufficient taxonomic resolution within particular lineages, as they target conserved regions shared among closely related taxa. Additionally, although Oxford Nanopore sequencing provides long reads, its higher per-base error rate may affect fine-scale sequence discrimination, potentially limiting species-level resolution (Delahaye and Nicolas 2021). Nevertheless, the ecological and sanitary relevance of the detected taxa should not be overlooked. The presence of these protozoans and fungal-related sequences in freshwater systems suggests that water may play a significant role in the transmission of infection to humans. Taken together, these findings emphasize the need for cautious interpretation and reinforce the importance of complementary molecular approaches to achieve reliable species-level resolution, which is essential for accurately assessing the zoonotic and anthroponotic risks associated with these microorganisms.

As a future perspective, methodological refinements could further strengthen the robustness of taxonomic and epidemiological inferences. The use of complementary higher-resolution molecular markers, such as *ITS2*, could help reduce error rates and improve the robustness of taxonomic assignments, although this approach may limit the ability to discriminate among closely related groups. In parallel, incorporating larger filtration volumes could enhance detection sensitivity by lowering the effective limit of detection (LOD), thereby increasing the probability of identifying microorganisms present at low abundances.

Our findings highlight the urgent need to strengthen water quality monitoring in regions facing environmental pressures from urban growth, tourism, and poor sanitation. The detection of microbial taxa of public health relevance, particularly in areas affected by untreated wastewater and seasonal runoff, underscores the risks to both human and ecosystem health. While NGS has proven effective for identifying potentially pathogenic microorganisms, further research is needed to understand their ecological dynamics and interactions with human and animal hosts. Integrating microbial data with epidemiological evidence will be key to developing effective One Health strategies in biodiverse and vulnerable regions like Santa Marta. Finally, amplicon-based sequencing of the 16S-rRNA and 18S-rRNA, when combined with traditional microbiological methods, offers valuable insights into microbial community structure and the influence of external factors on their composition and ecological function.

## Conclusions

This study demonstrates that the Gaira and Manzanares rivers, which are critical sources of water for domestic, agricultural, and recreational use in northern Colombia, exhibit significant microbial and physicochemical contamination, especially in downstream areas influenced by human settlements, tourism, and limited sanitation infrastructure. The detection of *Cryptosporidium* spp. by PCR highlights the presence of protozoan pathogens of public health relevance. NGS also enabled the broader characterization of microbial communities, revealing additional medically and environmentally relevant taxa, including enteric bacteria (*Escherichia-Shigella*), fungal genera, and other parasitic eukaryotes. These findings reflect the influence of anthropogenic pressures, especially in downstream areas, and underscore the limitations of relying solely on traditional or targeted approaches. The complementary use of NGS expands the scope of microbial detection, offering high-resolution insights into microbial diversity, potential pathogenic risks, and ecological gradients. This integrated strategy represents a valuable tool for enhancing water quality surveillance, particularly in biodiverse and vulnerable regions like Santa Marta, and supports the development of informed One Health interventions.

## Supplementary information

Below is the link to the electronic supplementary material.

ESM 1 Supplementary Fig. 1. Physicochemical variables evaluated: a) Total suspended solids (TSS), b) Turbidity, c) Nitrites, d) Nitrates, e) Phosphates, f) Ammonium, g) Biochemical oxygen demand (BOD), h) Chemical oxygen demand (COD), i) Conductivity, j) pH, k) Water Temperature and l) O2 at the sampling sites of the Gaira and Manzanares rivers (PNG 2.48 MB)

ESM 1 High Resolution Image (TIF 11.5 MB)

ESM 2 Supplementary Fig. 2 Rarefaction curves for the a) 16S-rRNA and b) 18S-rRNA datasets by site in each river, showing the number of species observed as a function of sample size (number of reads) (PNG 201 KB)

ESM 2 High Resolution Image (TIF 939 KB)

ESM 3 Supplementary Fig. 3. Alpha diversity indices for a) prokaryotic community (16S-rRNA) and b) eukaryotic community (18S-rRNA). Significant differences between upstream and downstream sites were detected only for the prokaryotic community in the Manzanares River, specifically for the Shannon (*p* = 0.022) and Simpson (*p* = 0.013) diversity indices (Wilcoxon rank-sum tests) (PNG 201 KB)

ESM 3 High Resolution Image (TIFF 2.41 MB)

ESM 4 Supplementary Fig. 4. Beta diversity Principal Coordinate Analysis (PCoA) based on dissimilarity of Bray-Curtis distances for a) prokaryotic community (16S-rRNA) and b) eukaryotic community (18S-rRNA) d) in the Gaira and Manzanares river. A significant interaction between basin and longitudinal position (upstream vs. downstream; *p* = 0.0117) was detected for the prokaryotic community (16S-rRNA). In contrast, river identity had a significant effect on community structure (*p* = 0.0488) for the eukaryotic community (18S-rRNA) (PNG 1.13 MB)

ESM 4 High Resolution Image (TIFF 7.84 MB)

ESM 5 (XLSX 47.6 KB)

## Data Availability

Raw sequencing data were deposited in NCBI GenBank under BioProject (PRJNA1235296).
