# Supplementary figures and images for: Assessment of water quality and microbial contamination in Santa Marta’s major rivers using conventional methods and next-generation sequencing
Source: Environ Sci Pollut Res Int. 2026 May 16;33(17):8335–49. doi: 10.1007/s11356-026-37784-y (PMC13226460; doi:10.1007/s11356-026-37784-y)

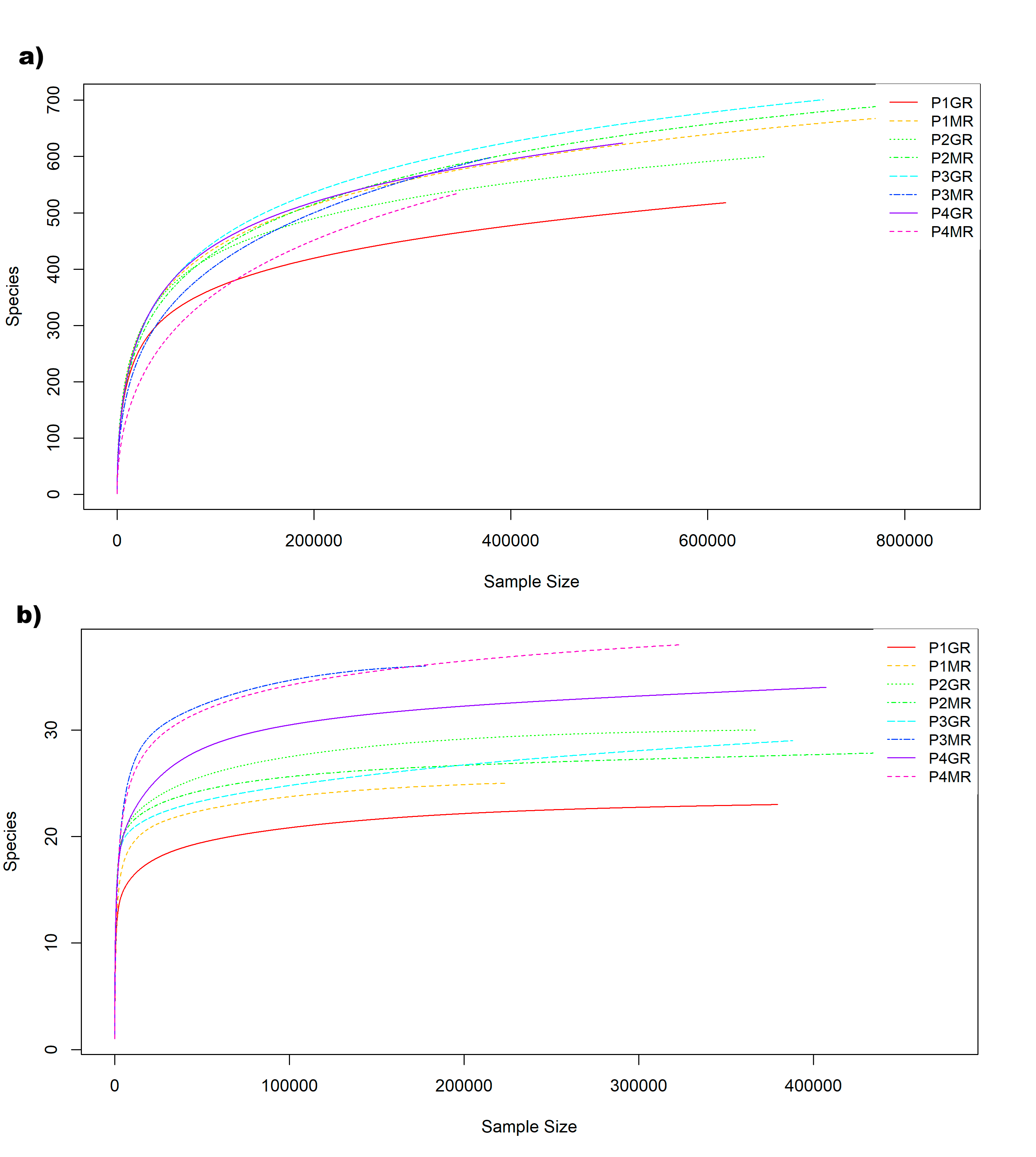

Supplement: Supplementary file 3 — Supplementary Fig. 2 Rarefaction curves for the a) 16S-rRNA and b) 18S-rRNA datasets by site in each river, showing the number of species observed as a function of sample size (number of reads) (PNG 201 KB) [file 11356_2026_37784_Fig5_ESM.png]

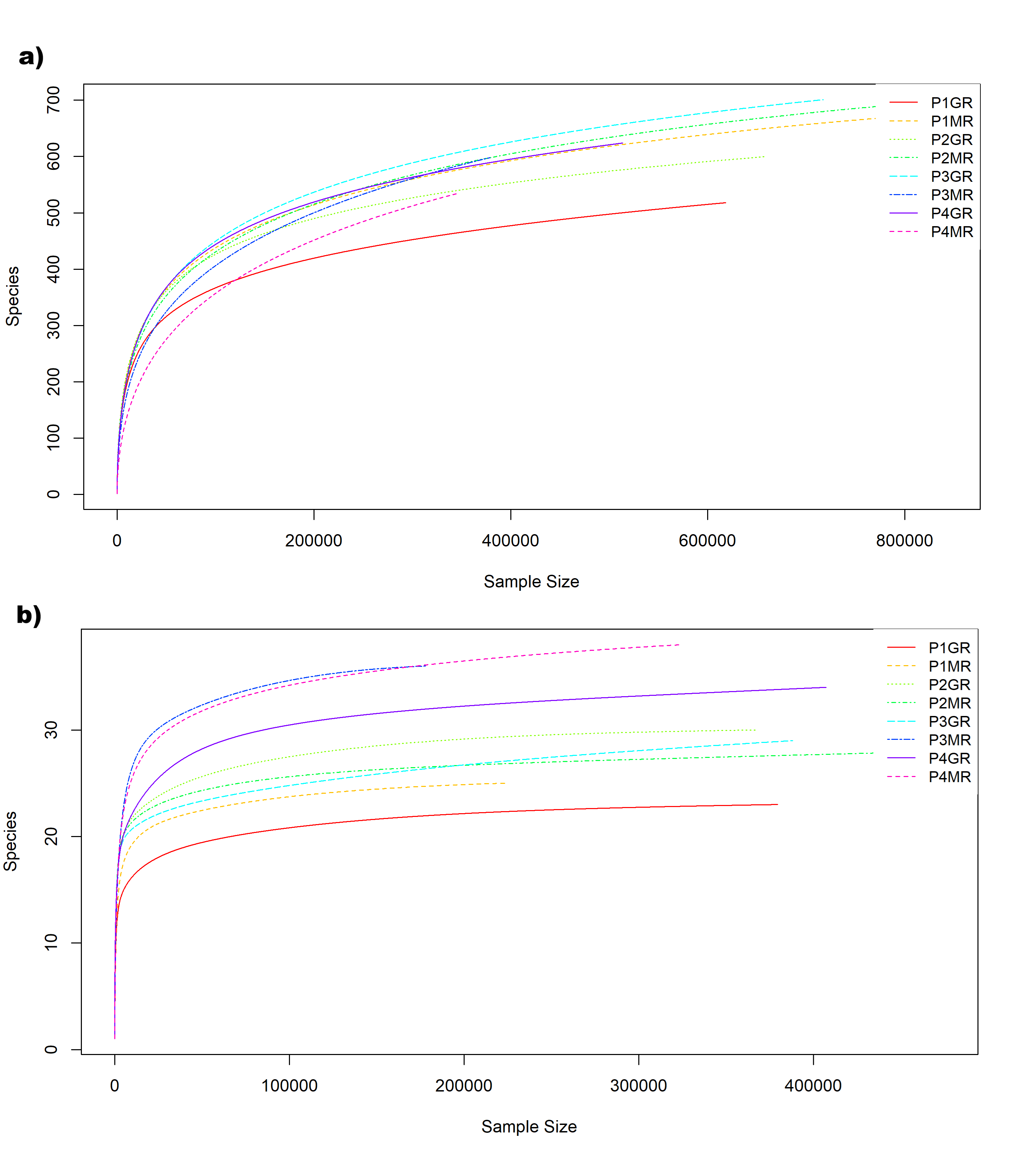

Supplement: Supplementary file 4 — High Resolution Image (TIF 939 KB) [file 11356_2026_37784_MOESM2_ESM.tif]

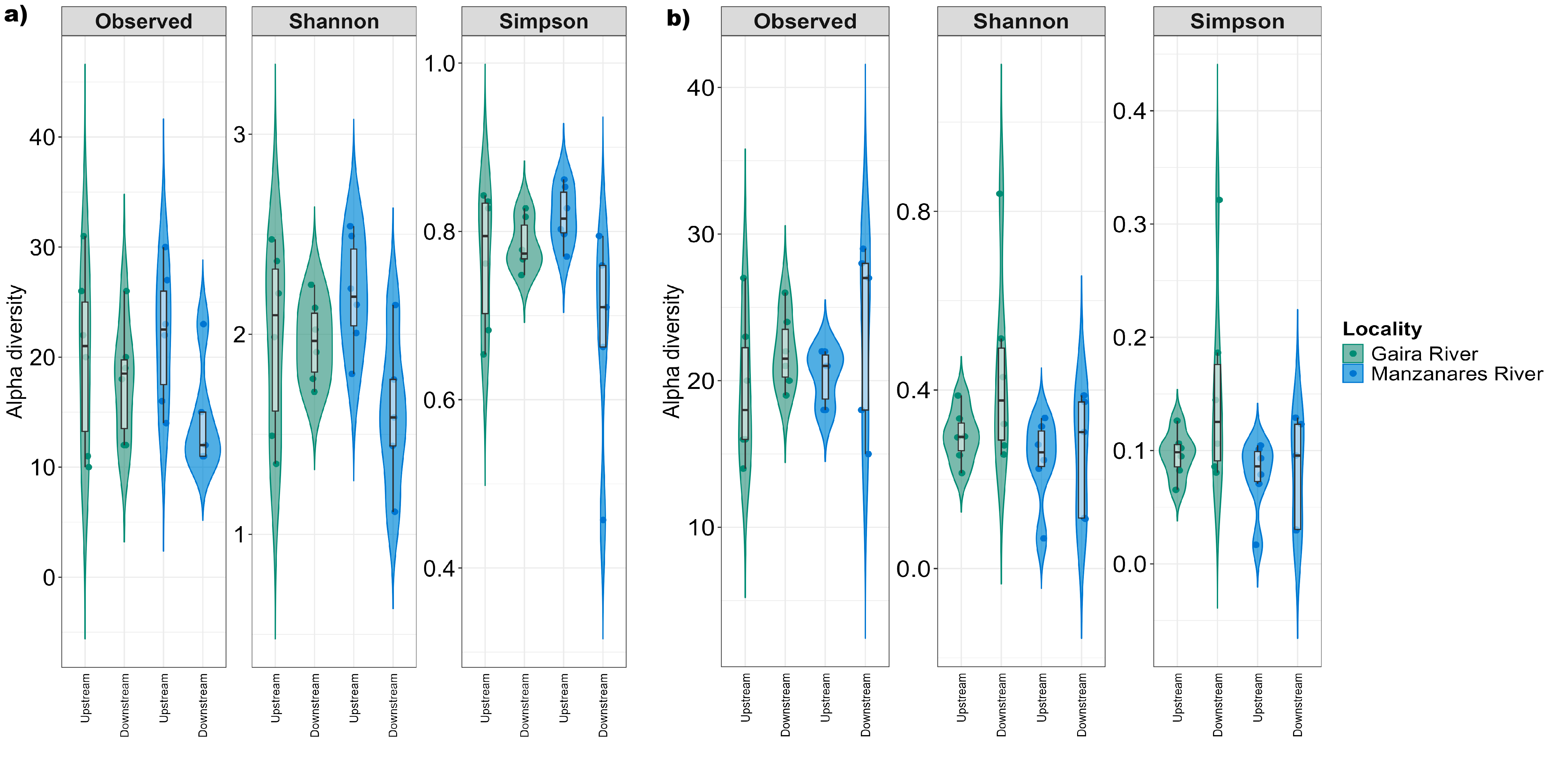

Supplement: Supplementary file 5 — Supplementary Fig. 3. Alpha diversity indices for a) prokaryotic community (16S-rRNA) and b) eukaryotic community (18S-rRNA). Significant differences between upstream and downstream sites were detected only for the prokaryotic community in the Manzanares River, specifically for the Shannon (p = 0.022) and Simpson (p = 0.013) diversity indices (Wilcoxon rank-sum tests) (PNG 201 KB) [file 11356_2026_37784_Fig6_ESM.png]

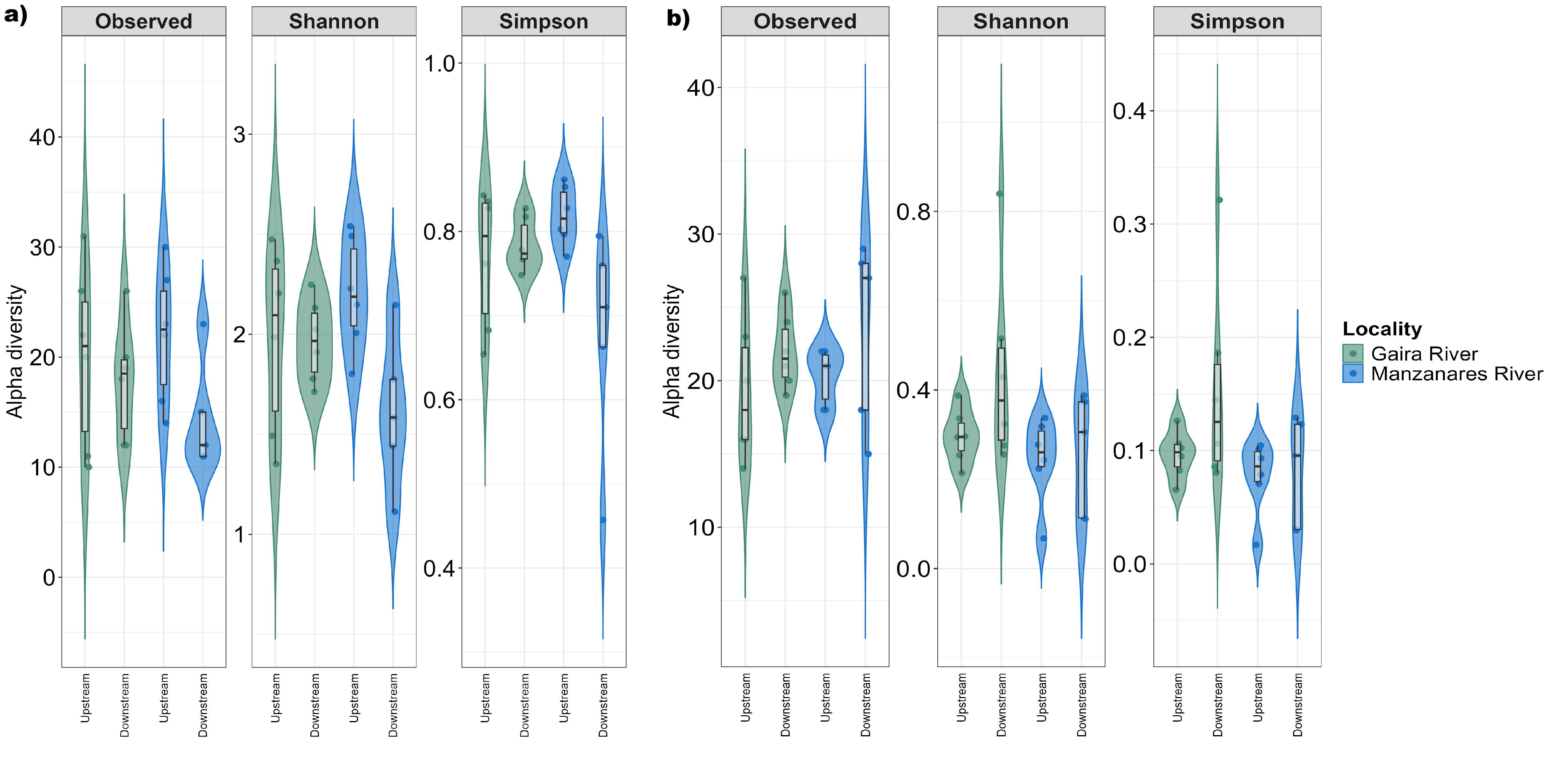

Supplement: Supplementary file 6 — High Resolution Image (TIFF 2.41 MB) [file 11356_2026_37784_MOESM3_ESM.tiff]

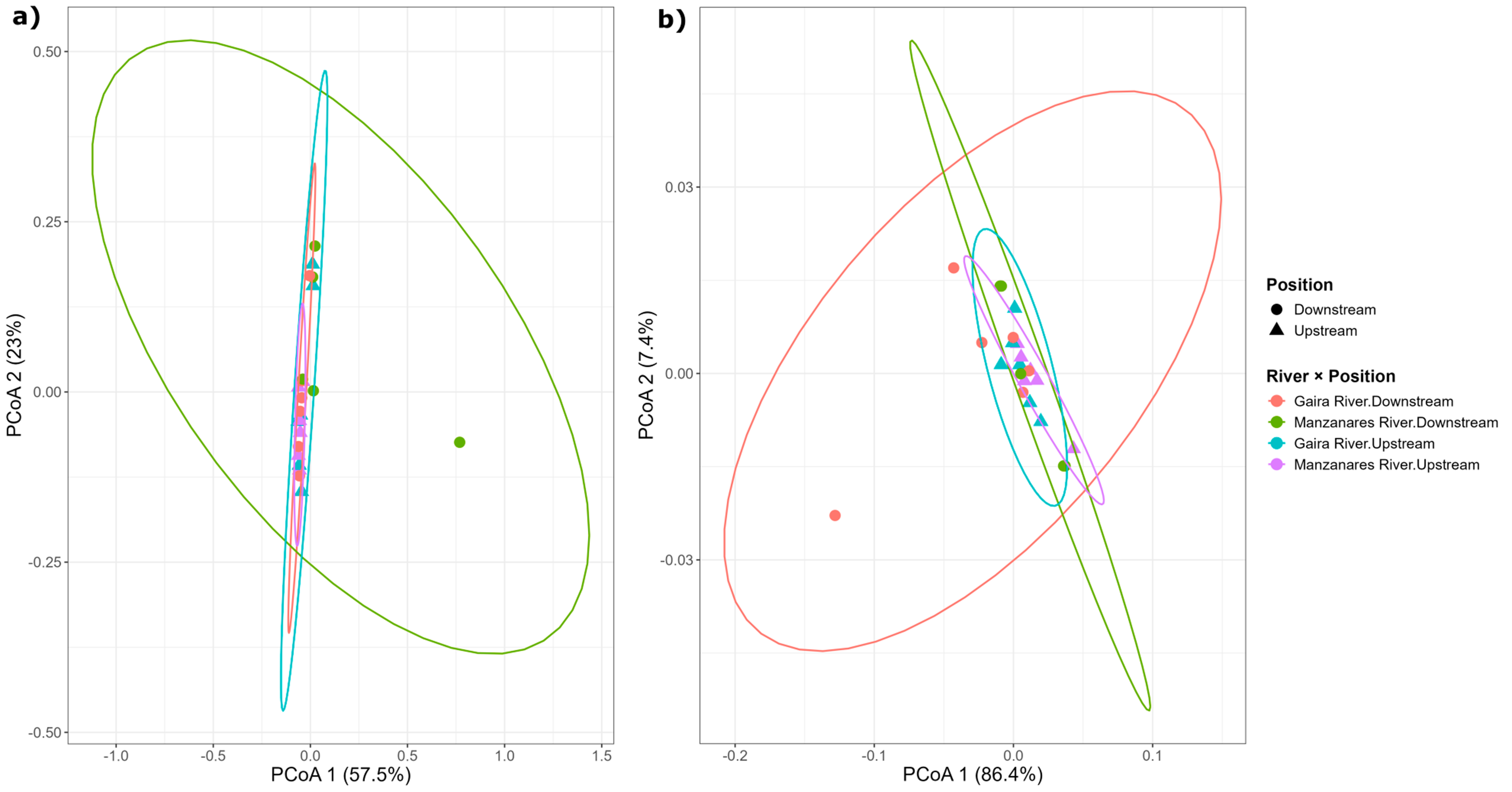

Supplement: Supplementary file 7 — Supplementary Fig. 4. Beta diversity Principal Coordinate Analysis (PCoA) based on dissimilarity of Bray-Curtis distances for a) prokaryotic community (16S-rRNA) and b) eukaryotic community (18S-rRNA) d) in the Gaira and Manzanares river. A significant interaction between basin and longitudinal position (upstream vs. downstream; p = 0.0117) was detected for the prokaryotic community (16S-rRNA). In contrast, river identity had a significant effect on community structure (p = 0.0488) for the eukaryotic community (18S-rRNA) (PNG 1.13 MB) [file 11356_2026_37784_Fig7_ESM.png]

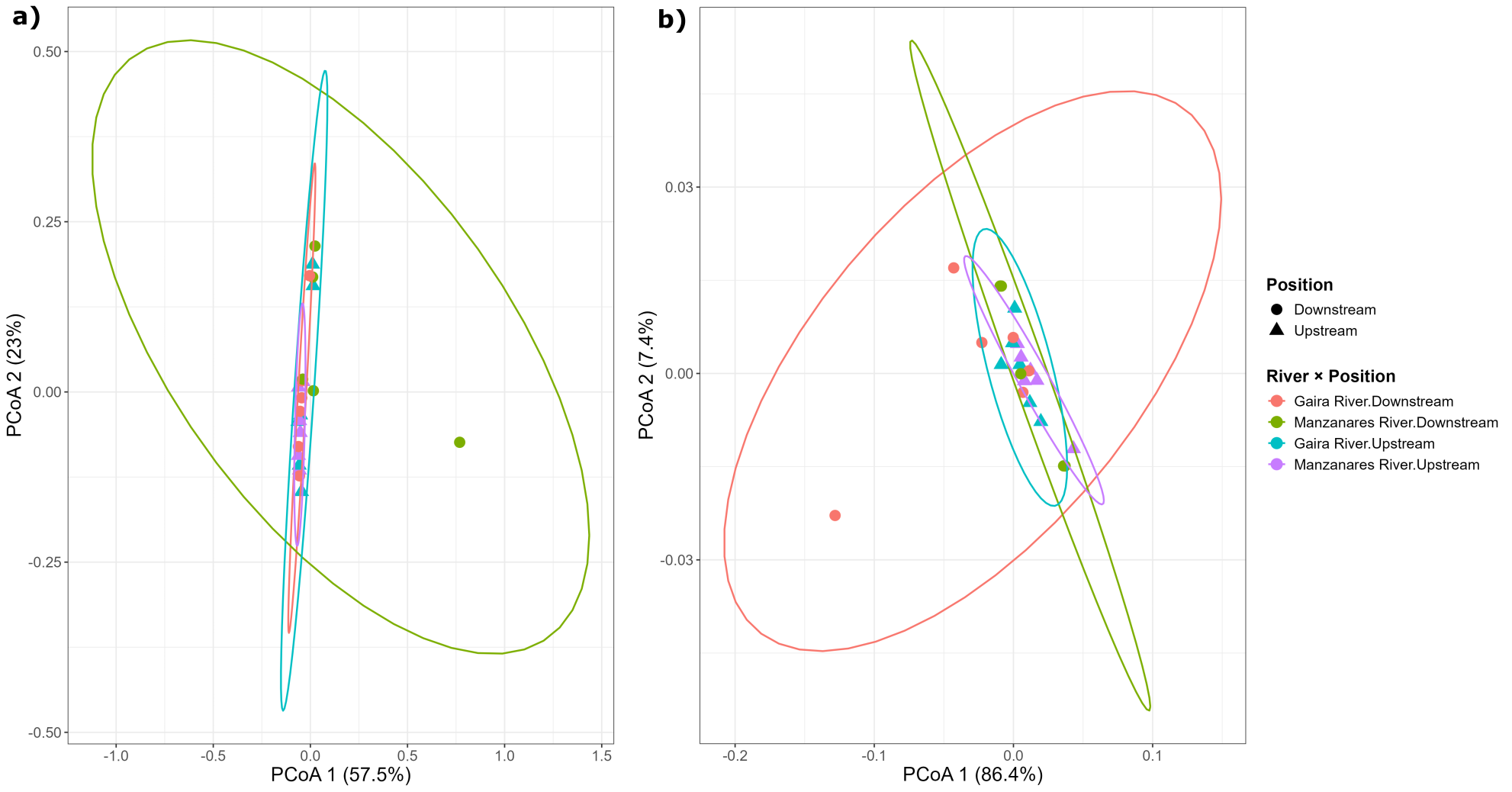

Supplement: Supplementary file 8 — High Resolution Image (TIFF 7.84 MB) [file 11356_2026_37784_MOESM4_ESM.tiff]
